# A *Tourist*-like MITE insertion in the upstream region of the *BnFLC.A10* gene is associated with vernalization requirement in rapeseed (*Brassica napus* L*.*)

**DOI:** 10.1186/1471-2229-12-238

**Published:** 2012-12-15

**Authors:** Jinna Hou, Yan Long, Harsh Raman, Xiaoxiao Zou, Jing Wang, Shutao Dai, Qinqin Xiao, Cong Li, Longjiang Fan, Bin Liu, Jinling Meng

**Affiliations:** 1 National Key Laboratory of Crop Genetic Improvement, Huazhong Agricultural University, Wuhan, 430070, China; 2 EH Graham Centre for Agricultural Innovation (an alliance between the Charles Sturt University and NSW Department of Primary Industries), Wagga Wagga Agricultural Institute, Wagga Wagga, NSW, 2650, Australia; 3 Department of Agronomy & James D. Watson Institute of Genome Sciences, Zhejiang University, Hangzhou, 310058, China; 4 Center of Systematic Genomics, Xinjiang Institute of Ecology and Geography, Chinese Academy of Sciences, Urumqi, 830011, China; 5 Key Laboratory of Experimental Marine Biology, Institute of Oceanology, Chinese Academy of Sciences, Qingdao, 266071, China

**Keywords:** Rapeseed, Flowering time, Vernalization, *Tourist*-like MITE, *FLOWERING LOCUS C*, Association analysis

## Abstract

**Background:**

Rapeseed (*Brassica napus* L*.*) has spring and winter genotypes adapted to different growing seasons. Winter genotypes do not flower before the onset of winter, thus leading to a longer vegetative growth period that promotes the accumulation and allocation of more resources to seed production. The development of winter genotypes enabled the rapeseed to spread rapidly from southern to northern Europe and other temperate regions of the world. The molecular basis underlying the evolutionary transition from spring- to winter- type rapeseed is not known, however, and needs to be elucidated.

**Results:**

We fine-mapped the spring environment specific quantitative trait locus (QTL) for flowering time, *qFT10-4*,in a doubled haploid (DH) mapping population of rapeseed derived from a cross between Tapidor (winter-type) and Ningyou7 (semi-winter) and delimited the *qFT10-4* to an 80-kb region on chromosome A10 of *B. napus*. The *BnFLC.A10* gene, an ortholog of *FLOWERING LOCUS C* (*FLC*) in *Arabidopsis*, was cloned from the QTL. We identified 12 polymorphic sites between *BnFLC.A10* parental alleles of the TN-DH population in the upstream region and in intron 1. Expression of both *BnFLC.A10* alleles decreased during vernalization, but decreased more slowly in the winter parent Tapidor. Haplotyping and association analysis showed that one of the polymorphic sites upstream of *BnFLC.A10* is strongly associated with the vernalization requirement of rapeseed (r^*2*^ = 0.93, *χ*^*2*^ = 0.50). This polymorphic site is derived from a *Tourist*-like miniature inverted-repeat transposable element (MITE) insertion/deletion in the upstream region of *BnFLC.A10*. The MITE sequence was not present in the *BnFLC.A10* gene in spring-type rapeseed, nor in ancestral ‘A’ genome species *B. rapa* genotypes. Our results suggest that the insertion may have occurred in winter rapeseed after *B. napus* speciation.

**Conclusions:**

Our findings strongly suggest that (i) *BnFLC.A10* is the gene underlying *qFT10-4*, the QTL for phenotypic diversity of flowering time in the TN-DH population, (ii) the allelic diversity caused by MITE insertion/deletion upstream of *BnFLC.A10* is one of the major causes of differentiation of winter and spring genotypes in rapeseed and (iii) winter rapeseed has evolved from spring genotypes through selection pressure at the *BnFLC.A10* locus, enabling expanded cultivation of rapeseed along the route of *Brassica* domestication.

## Background

Interaction between various environmental signals and flowering genes is critical for plants to flower and complete their life cycle, and thus important to humans, who rely upon adequate production of fruit and seeds to feed the world’s growing population. Climate change fluctuations accompanying global warming [1,2] are requiring plant breeders to elucidate the molecular mechanisms underlying flowering, and to develop strategies for manipulating and optimizing the flowering times to maximize crop yields. Four flowering pathways—autonomous, vernalization, photoperiod and gibberellic acid—have been established in *Arabidopsis* and partially identified in other species [3,4]. Vernalization is an adaptive trait in which plants acquire the ability to flower following exposure to cold temperatures. A series of genes in the endogenous network involved in this process, and their regulatory relationships, have been identified; genes from different flowering pathways function together with other integrator genes to control flowering [5,6]. The MADS-box family gene *FLOWERING LOCUS C* (*FLC*) represses flowering [7,8] by suppressing the expression of *FLOWERING LOCUS T* (*FT*), a key flowering integrator and confirmed florigen in plants [9-11], and other floral integrator genes such as *FLOWERING DURATION* and *SUPPRESSOR OF OVEREXPRESSION OF CONSTANS*[9,12,13]. Expression of *FLC* is reduced by vernalization [7,8]. *FLC* is up-regulated by *FRIGIDA* (*FRI*) and repressed by genes in the autonomous pathway [14-16]. *FLC* expression has also been shown to be regulated via histone acetylation and methylation, which alters the expression of a trans-acting regulator common to *FLC* and members of the related *MADS AFFECTING FLOWERING* gene [17-20].

The genus *Brassica*, which diverged from *Arabidopsis* 14.5 to 20.4 million years ago [21-23] includes more crops of agricultural and horticultural importance than any other genus in the family of Brassicaceae. Comparative analysis has revealed that diploid *Brassica* genomes are composed of conserved segments triplicated from *Arabidopsis*[24,25]. The allopolyploid species *B. napus* (rapeseed, oilseed rape or canola; genomes AACC, 2*n* = 4x = 38) is a product of natural hybridization between diploid species *B. rapa* (2*n* = 2x = 20, genome AA) and *B. oleracea* (2n = 2x =18, genome CC). Rapeseed originated in southern Europe along the coastline of the Mediterranean Sea 10,000–100,000 years ago, and was domesticated as an oil crop 400–500 years ago. This crop was originally grown as a spring or semi-winter crop in Mediterranean climates; Its cultivation spread rapidly from southern into northern Europe after the development of winter rapeseed varieties, which do not flower during the long and cold winters. Understanding the evolution of flowering time is critical for domestication and introduction of rapeseed into new agroclimatic regions.

Miniature inverted-repeat transposable elements (MITEs) belong to a class of non-autonomous DNA transposable elements known as class II transposons. They are present in high copy number in the genome and contribute to genomic structure variations and intra-species diversity [26,27]. Differing MITE insertion profiles among varieties of a given species enable tolerance to environmental changes and allow adaptation under selective pressure [26,28,29].

Genetic analyses of several mapping populations of *Brassica* have revealed that both major and minor quantitative trait loci (QTLs) control flowering time. Some of these QTLs have also been shown to collocated with candidate genes for flowering time such as *CO, FLC, FT* and *FRI*[30-35]. Forty-two QTLs were identified in a doubled haploid (DH) rapeseed mapping population (TN DH) derived from a cross between Tapidor and Ningyou7, but their magnitude and genetic effects varied with growing environment [36]. One major flowering time QTL, *qFT10-4*, which accounted for more than 50% of phenotypic variation in flowering time in the TN DH populations grown in non-vernalization environments, was colocalized with the ortholog of *FLC* from *Arabidopsis* in chromosome A10 and was designated *BnFLC.A10*[36,37]. In our study, the candidate gene *BnFLC.A10* for *qFT10-4* was dissected using a map-based cloning approach, and an association was found between a *Tourist*-like MITE insertion/deletion in the upstream region of *BnFLC.A10* and the stronger vernalization requirement in rapeseed.

## Results

### Cloning of *BnFLC.A10* from *qFT10-4* and allelic divergence

**Figure 1:**
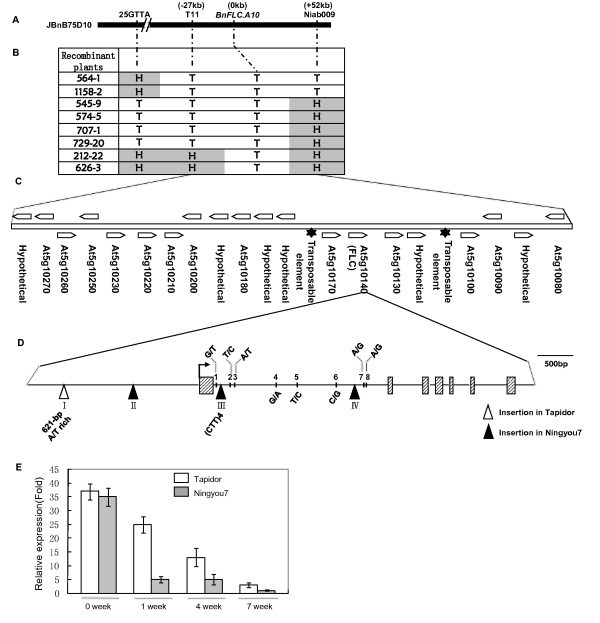
**Cloning of *qFT10-4 *and detailed structure and allelic divergence of *BnFLC.A10*.** (**A**) Positions of markers used to fine-map *qFT10-4* are shown in the BAC clone JBnB75D10 of *B. napus* ‘Tapidor’. Marker IP1IP2 was developed from a specific sequence of *BnFLC.A10*. (**B**) Genotypes of recombinants detected among non-flowering plants of the BC_5_F_2_ segregation population derived from the TN DH line DH043 (winter-type) and Ningyou7 (semi-winter-type). T and H represent homozygous and heterozygous genotypes, respectively, for the Tapidor allele. (**C**) Genes identified in the 80-kb region of JBnB75D10 that was delimited with markers T11 and Niab009. Arrows show the relative positions of predicted open reading frames (ORFs). For each ORF, the orthologous gene in *A. thaliana* is marked and genes that lacked an ortholog are labelled ‘hypothetical’. (**D**) Schematic diagram of the DNA sequence of *BnFLC.A10*. The arrow shows the translation start site. Roman numerals indicate the indels (I–IV) between the alleles from Tapidor and Ningyou7. Vertical bars labeled with Arabic numerals represent SNPs (1–8). For the SNPs, the nucleotide found in the Tapidor allele is given first. (**E**) *BnFLC.A10* expression as detected by quantitative PCR during different stages of vernalization (0 to 7 weeks) at 4°C. Expression of the Ningyou7 allele decreased much more rapidly than that of the Tapidor allele during vernalization.

To construct a high-resolution map of the *qFT10-4* locus, we analyzed a large BC_5_F_2_ population (9,000 plants) that was derived from a cross between the DH line TN DH043 (winter-type) and Ningyou7 (semi-winter-type). Four molecular markers developed from the sequence of the Bacterial Artificial Chromosome (BAC) clone JBnB75D10, which contains *BnFLC.A10*, were used for the analysis (Figure 1A). Eight recombinants were identified and the QTL *qFT10-4* was delimited to an 80-kb region that showed collinearity with the top of chromosome 5 of *Arabidopsis thaliana* (Figure 1B and C). None of the genes in this region except *FLC* are known to be involved in floral transition.

To analyze the basis of the vernalization requirement in rapeseed, we cloned and compared *BnFLC.A10* sequences (approximately 7 kb) from Tapidor and Ningyou7, the parental lines of the mapping population. No polymorphism was found in the coding sequence (CDS) between the two alleles (*BnFLC.A10-T* and *BnFLC.A10-N*). However, there were two insertion/deletions (indels I and II) in the upstream region, together with two indels (indels III and IV) and eight single nucleotide polymorphisms (SNPs 1–8) in intron 1 of *BnFLC.A10* (Figure 1D). Expression analysis showed that *BnFLC.A10-N* was markedly down-regulated upon exposure to cold treatment after 1 week, whereas expression of *BnFLC.A10-T* decreased gradually over 7 weeks of cold treatment (Figure 1E). This observation provides strong evidence that *BnFLC.A10* underlies variation for vernalization requirement and that differences in gene expression establish the basis for allelic variation at the *qFT10-4* locus.

### A 621-bp insertion upstream of *BnFLC.A10-T* is associated with winter habit in rapeseed

**Figure 2:**
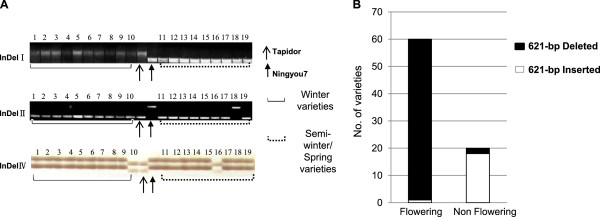
**Association of polymorphic sites (indels and SNPs) in *BnFLC.A10 *and flowering phenotype in rapeseed cultivars that were planted in spring.** (**A**) Genotyping of indels by PCR in some of the analyzed cultivars. Because the polymorphic products for indel III could not be distinguished by PCR, results for this indel are not shown. Lanes 1–19 represent the corresponding PCR products amplified from the genomic DNA of accessions of Apache, Bakow, Bienvenu, Bolko, Brutor, Casino, Ceres, Diadem, JeT-Neuf, Coma, Apomix, Chuanyou11, Dac-chosen, Dunkeld, Erglu, Huashuang2, Jiayou1, Huáyou2 and Karoo. For detailed genotypic information, see Table 1. (**B**) Association between indel I and flowering phenotype.

To determine whether sequence variations in the two *BnFLC.A10* alleles contribute to differences in vernalization requirements or winter growth habit among natural rapeseed populations, we conducted an association analysis using a panel of 79 diverse rapeseed cultivars representing winter, semi-winter and spring genotypes. All of the cultivars were planted in spring environments. Because of lack of vernalization, none of the winter-type cultivars flowered; in contrast all of the spring-type and semi-winter-type cultivars (with one exception) flowered normally (Table 1). The three largest indels (I, II and IV) were analyzed first because the alleles could be easily distinguished by PCR (Figure 2A). For indel I, the 621-bp insertion was absent in all spring and semi-winter cultivars, but was present in all 18 winter cultivars except Coma. The 621-bp insertion showed a highly significant correlation with flowering phenotype (r^*2*^ = 0.93, Table 2; Figure 2B). In contrast, Indel II (r^*2*^ = 0.49) and indel IV (r^*2*^ = 0.56) were only weakly associated with flowering phenotype (Table 2).

**Table 1:** **Phenotypic and genotypic data for 79 *B. napus *accessions**

**Accession**	**Origin**	**Type**	**Genotype**^**a**^			**Days to flowering**		
			**InDel I**	**InDel II**	**InDel IV**	**2007**	**2008**	**2009**
Altex	Canada	Spring	N	T	T	69	61	64
Alto	Canada		N	T	T	65	61	61
Apomix	Unknown		N	N	N	65	61	61
Bronowski DH2	Poland		N	N	N	89	85	87
Bullet	Canada		N	T	T	67	61	63
Celebra	Unknown		N	N	N	72	72	75
CENN	Unknown		N	N	N	78	73	77
Comet	Denmark		N	T	T	75	68	68
Conzuul	Unknown		N	T	-	74	68	69
D.ARoll	Unknown		N	T	T	72	67	67
Dac-chosen	Unknown		N	N	N	67	67	67
Dunkeld	Australia		N	N	N	71	71	73
Erglu	Germany		N	N	N	76	64	66
Erra	Germany		N	N	N	69	70	72
Global	Canada		N	T	T	78	64	66
Granit	Sweden		N	T	T	79	59	62
Grouse	Australia		N	T	-	69	60	57
GULLR	Sweden		N	T	T	78	73	73
Jiayou1	Canada		N	N	N	76	91	78
Jiayou3	Canada		N	N	N	75	77	74
Karoo	Australia		N	N	N	65	58	66
Marnoo	Australia		N	N	N	69	65	67
Monty	Australia		N	T	T	69	56	60
Niklas	Sweden		N	T	T	78	67	72
Nilla	Sweden		N	T	T	82	77	73
Ning RS-1	China		N	N	-	83	79	81
Qingyou2	China		N	N	N	65	60	63
Rioklas	Unknown		N	T	T	75	73	74
Rucabo	Germany		N	N	N	74	68	69
Westar	Canada		N	T	T	50	50	50
Chuanyou11	China	Semi-winter	N	N	N	67	71	72
Fuyou1	China		N	N	N	66	69	67
Fuyou2	China		N	T	H	67	65	65
Gànyou14	China		N	T	H	75	77	76
Gānyou2	China		N	N	N	67	77	77
Gànyou3	China		N	N	N	69	67	68
Gānyou5	China		N	N	N	67	62	63
Huashuang1	China		N	N	N	72	72	73
Huashuang2	China		N	N	T	81	82	78
Huashuang3	China		N	T	T	75	72	68
Huáyou10	China		N	N	N	69	69	69
Huáyou11	China		N	N	N	68	68	68
Huáyou12	China		N	N	N	69	69	69
Huáyou13	China		N	N	N	72	72	72
Huáyou14	China		N	N	N	71	71	71
Huáyou16	China		N	N	-	72	76	79
Huáyou2	China		N	T	N	67	67	73
Huāyou3	China		N	N	N	74	74	74
Huāyou4	China		N	N	N	63	63	63
Huāyou6	China		N	N	N	67	67	67
Huāyou9	China		N	N	N	NF	NF	NF
huāyuo5	China		N	T	T	69	68	62
Huáyuo6	China		N	T	-	61	68	72
Suyou3	China		N	N	N	62	59	59
Xiangnongyou2	China		N	N	N	70	70	70
Xiangnongyou3	China		N	N	N	67	67	67
Xiangyou13	China		N	N	N	70	73	74
Youyan2	China		N	N	N	64	60	61
Zhenyou-1	China		N	T	H	67	78	80
Zheyou7	China		N	N	N	72	67	69
Apache	UK	Winter	T	T	T	NF	NF	NF
Bakow	Poland		T	T	T	NF	NF	NF
Bienvenu	France		T	T	T	NF	NF	NF
Bolko	Poland		T	T	T	NF	NF	NF
Brutor	France		T	T	T	NF	NF	NF
Casino	Sweden		T	T	T	NF	NF	NF
Ceres	Germany		T	T	T	NF	NF	NF
Coma	Unknown		N	T	H	NF	NF	NF
D-083	Unknown		T	T	T	NF	NF	NF
Diadem	Germany		T	T	T	NF	NF	NF
JeT-Neuf	France		T	T	T	NF	NF	NF
Jupiter	Sweden		T	T	T	NF	NF	NF
Libritta	Germany		T	T	T	NF	NF	NF
Liradonna	Germany		T	T	T	NF	NF	NF
Literavo	Germany		T	T	T	NF	NF	NF
Matador	Sweden		T	T	T	NF	NF	NF
Nestor	Sweden		T	T	T	NF	NF	NF
Panter	Sweden		T	-	T	NF	NF	NF
Quinta	Germany		T	T	T	NF	NF	NF

^a^T, N and H indicate homozygous for Tapidor, homozygous for Ningyou7 and heterozygous genotypes, respectively; - represents an undetectable genotype or a new genotype distinct from the two parents.

The gene.

**Table 2:** **Haplotypes detected with the sequence information of *BnFLC.A10 *from 24 *B. napus *accessions**

**Haplotype**	**Accession**	**Origin**	**Type**	**InDel**				**SNP**						**Day to flowering**
				I^***^	II^*^	III^a^	IV^*^	1^*^	2^*^	3^*^	4	5	6	
HapI	Tapidor	France	Winter	In	DEL	6	DEL	G	T	A	G	G	A	Non-flowering
	Apache	UK	Winter	In	DEL	6	DEL	G	T	A	G	G	A	Non-flowering
	Bakow	Poland	Winter	In	DEL	6	DEL	G	T	A	G	G	A	Non-flowering
	Bienvenu	France	Winter	In	DEL	6	DEL	G	T	A	G	G	A	Non-flowering
	Brutor	France	Winter	In	DEL	6	DEL	G	T	A	G	G	A	Non-flowering
	Casino	Sweden	Winter	In	DEL	6	DEL	G	T	A	G	G	A	Non-flowering
	Quinta	Germany	Winter	In	DEL	6	DEL	G	T	A	G	G	A	Non-flowering
HapII	Alto	Canada	Spring	DEL	DEL	6	DEL	G	T	A	G	G	A	62
	Bullet	Canada	Spring	DEL	DEL	6	DEL	G	T	A	G	G	A	64
	Comet	Denmark	Spring	DEL	DEL	6	DEL	G	T	A	G	G	A	70
	GULLR	Sweden	Spring	DEL	DEL	6	DEL	G	T	A	G	G	A	75
	Westar	Canada	Spring	DEL	DEL	6	DEL	G	T	A	G	G	A	50
HapIII	Qingyou2	China	Spring	DEL	In	6	DEL	G	T	A	G	G	A	63
HapIV	Erglu	Australia	Spring	DEL	In	7	In	A	C	T	G	G	A	69
HapV	Gānyou5	China	Semi-winter	DEL	In	7	In	A	C	T	A	C	G	64
	Huashuang1	China	Semi-winter	DEL	In	7	In	A	C	T	A	C	G	72
	Huāyou4	China	Semi-winter	DEL	In	7	In	A	C	T	A	C	G	63
	Karoo	Australia	Spring	DEL	In	7	In	A	C	T	A	C	G	63
	Suyou3	China	Semi-winter	DEL	In	7	In	A	C	T	A	C	G	60
	Xiangyou13	China	Semi-winter	DEL	In	7	In	A	C	T	A	C	G	72
HapVI	Erra	Germany	Spring	DEL	In	10	In	A	C	T	A	C	G	70
	Jiayou1	China	Spring	DEL	In	10	In	A	C	T	A	C	G	82
	NingRS-1	China	Semi-winter	DEL	In	10	In	A	C	T	A	C	G	81
	Ningyou7	China	Semi-winter	DEL	In	10	In	A	C	T	A	C	G	72

Symbols * and *** represent p = 0.05 and 0.001 levels of significance of each site’s influence on flowering time.

^a^The repeats of the CTT motif in indel III; In = insertion; DEL = deletion.

Haplotyping of *BnFLC.A10* specific markers for indels I–IV and SNPs 1–6 (Figure 1D) confirmed that most winter rapeseeds had a 621-bp insertion in the upstream region of *BnFLC.A10* (haplotype I), whereas the 621-bp fragment was absent in the spring types (Table 2). These results suggest that indel I (with the 621-bp fragment present or absent) in the upstream region of *BnFLC.A10* plays a very important role in modulating flowering time in natural rapeseed germplasm and potential development of a winter growth habit.

### The 621-bp insertion in the upstream region of *BnFLC.A10* is a *Tourist*-like MITE

**Figure 3:**
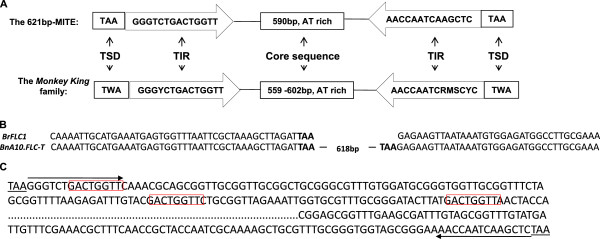
**Structure of the 621-bp MITE and its family.** (**A**) Basic structure of the 621-bp MITE and elements in the *Monkey King* family. The consensus sequences of the TIRs and TSDs are shown. The length and content of the core AT-rich sequence varied among homologs of *Monkey King*. The numbers marked in the frame of core sequences represent the length (without TAAs and TSDs) of *Monkey King* upstream of *BnFLC.A10* and its homologs in the *B. rapa* genome. W=(A/T), Y=(C/T), M=(A/C), R=(A/G), and S=(C/G). (**B**) Flanking sequence of *Monkey King* at the 5’ upstream end of *BnFLC.A10-T* and the corresponding Related Empty Sites (RESites) in *B.rapa*. (**C**) Three replicates of the GACTGGTT motif scattered near the 5’ end region of *Monkey King*. The sequence of *Monkey King* is shown, with dots representing omitted portions. TSDs are underlined and TIRs are marked with arrows. GACTGGTT motifs are framed in red; all of the three duplications are located near the 5’ end region.

To further characterize the 621-bp insertion sequence in winter-type rapeseed accessions, *BnFLC.A10* alleles from the eight cultivars that representing haplotype I (Table 2) were sequenced and aligned. All the sequenced genotypes showed 100% identity. The inserted sequence possessed typical characteristics of a *Tourist*-like MITE [38-40], with 14-bp terminal inverted repeat (TIR) sequences flanked by target sequence duplications (TSDs) of TAA (Figure 3A). Between the TIR sequences, an AT-rich (67%) core that contained 12 classes of important motifs (such as the TATA box and CAAT box) was identified (Additional file 1). These motifs might function in transcriptional initiation or promotion, or in response to different stimuli and signals (Additional file 1). At least four homologs of the MITE insertion (BLASTN expected value < 1e^-10^) were identified in the genomic sequence of *B. napus* from public databases (http://www.ncbi.nlm.nih.gov) and up to 200 copies (E-value < 1e-^10^) were identified in the sequenced genome of *B. rapa*, the ancestral source of the A genome in *B. napus*. These homologs defined a new family of MITEs, which we named *Monkey King* (Figure 3B) after the subject of a Chinese myth. (In “Journey to the west”, *Monkey King* is capable of 72 methods of transformation and can transform hundreds of monkeys with one of his hairs. He also jumps long distances with a cloud somersault). 

### Origin and transmission of the *BnFLC.A10* 621-bp insertion in *B. napus* and its A genome ancestral species *B. rapa*

To understand the evolutionary process behind the adaptation associated with the insertion of *Monkey King* into the upstream region of *BnFLC.A10* and to trace its origin and transmission, we investigated an additional 154 spring cultivars of *B. napus* and 103 cultivars (including the genome sequenced cultivar, Chiifu [41]) belonging to nine subspecies of *B. rapa* (oilseed, swede and fodder types, Additional file 2). No *Monkey King* insertion was detected in the upstream region of *BnFLC.A10* in any of the accessions, even though the empty site of insertion was almost 100% identical to the sequences that flanking the *Monkey King* insertion in *BnFLC.A10* in winter rapeseed (Figure 3B and Additional file 2). On the other hand, hundreds of copies of *Monkey King* were detected in the whole genome, but not in the *BrFLC.A10* upstream region of *B. rapa* ‘Chiifu’. This suggests that *Monkey King* may have pre-existed in the *B. rapa* genome but was inactive, after the generation of *B. napus*, it was activated and inserted into the upstream region of *BnFLC.A10*, giving rise to winter rapeseed.

## Discussion

In this study, we used positional cloning to dissect the major flowering time QTL, *qFT10-4*, which was detected only in the spring-cropped TN DH population. The QTL *qFT10-4* on chromosome A10 was delimited in a narrow 80-kb genomic region and annotation of different genes allowed us to identify *BnFLC.A10*, an ortholog of *FLC*, as the candidate gene. We demonstrated for the first time that flowering time variation at the *qFT.10-4* locus is conditioned by the major vernalization response gene, *BnFLC.A10*; the MITE insertion upstream of *BnFLC.A10* show significant association with the flowering time variation between winter and spring rapeseed*.*

Control of flowering time by vernalization has previously been shown to depend on a complex regulatory network, especially in amphidiploid rapeseed. In one study of the relationship between flowering time and *FLC* orthologs, five *BnFLC* sequences were isolated from *B. napus* cDNA library and in another study six *FLC* paralogs have been identified in *B. napus* by comparative analysis of *B. napus* and *Arabidopsis* genomes [36,42]. The fact that indel I in the upstream region of *BnFLC.A10* cosegregated with flowering phenotype in the TN DH population but in only some of the diverse cultivars might be due to the contribution of other flowering time QTLs, including other *BnFLC*s, with very small genetic effects. For examples, one of the *BnFLC* paralogs, which was located in linkage group A3 (*BnFLC.A3b*), colocalized with the flowering time QTL and thus might contribute to the vernalization response in certain cultivars [43]. In fact, at least nine copies of *Bn.FLC* genes exist in rapeseed [43]. Other genes, such as *FRIGIDA*, also regulate *FLC* expression in rapeseed; *BnaA.FRI.a*, one of orthologs of *FRIGIDA* in *Arabidopsis*, contributes to flowering time variation in rapeseed, which may partly explain why indel I did not totally cosegregated with flowering time in the association analysis [33]. In our research, the cultivar “Coma” that lacked the *BnFLC.A10* upstream MITE insertion still exhibited the winter characteristic. It is thus possible that other copies of *BnFLC*s or related genes from the vernalization pathway may contribute to vernalization response in *B. napus*. The expression of one of the other *BnFLC* copies, or of all *BnFLC* copies acting in concert in the Coma genome, may be sufficient to inhibit flowering transition under spring environmental conditions, therefore enabling the cultivar ‘Coma’ to function as a winter rapeseed. Genetic diversity with respect to *BnFLC*s and other *Arabidopsis* vernalization pathway gene homologs has been associated with vernalization in *B. napus*, but no gene or polymorphic site as strongly associated with vernalization requirement of rapeseed as the *BnFLC.A10* upstream MITE insertion has been dissected previously. The MITE insertion into *BnFLC.A10* appears to be one of the most important causative factors of vernalization requirement in winter rapeseed cultivars.

It is believed that rapeseed originated from a natural hybridization between *B. rapa* and *B. oleracea* that occurred in southern Europe along the Mediterranean coastline approximately 10,000–100,000 years ago. Given the warm climate in this region year-round, naturally occurring rapeseed genotypes and their ancestors may not have needed to develop an adaptation requiring prolonged vernalization. The activation of *Monkey King* in *B. napus* genome would have introduced diversity into the germplasm upon which selective pressure could act. The insertion of *Monkey King* in the upstream region of *BnFLC.A10* resulted in strong dependence on vernalization for flowering; this characteristic was then selected by plant breeders during the development of winter-type rapeseed cultivars of rapeseed that could be grown in northern Europe and other temperate regions of the world.

Several studies have examined the effect of MITEs on neighboring gene expression. For example, the DNA methylation level of a MITE can influence expression of neighboring genes. An assay of transient and stably-transformed rice revealed that the MITE *Kiddo*, when present in the promoter of the rice *ubiquitin2* (*rubq2*) gene, was responsible for up to 20% of neighboring gene expression; most notably, when DNA methylation of *Kiddo* was blocked, transcript levels of endogenous *rubq2* increased threefold [44]. An association has also been reported between a MITE inserted in the upstream regulator region of the gene *Vgt1* (Vegetative to generative transition) and early flowering in Northern maize genotypes [45,46]. In our study, the MITE upstream of *BnFLC.A10* was positively associated with gene expression and induced *BnFLC.A10* expression during vernalization. The inserted MITE seems to attenuate cold-induced *BnFLC.A10* repression rather than increase its expression, in winter rapeseed. This result is very similar to that observed in *Arabidopsis*, where *FLC* expression was correlated with flowering time and vernalization requirement in unvernalized or long days, but not as strongly as anticipated [47,48]. We thus conclude that either decreased rate of *FLC* expression during vernalization or additional epistatic interaction with other genes is more important for control of flowering time and vernalization requirement than variation of *FLC* expression under unvernalized conditions. Using motif prediction, motifs associated with gene regulation were found to exist in the *Monkey King* sequence (Additional file 1). Most of these motifs were located in gene promoter and enhancer regions (TATA box and CAAT box) or were light responsive elements (Sp1) (Additional file 1) associated with response to environmental signals in different organisms. Certain transcriptional factors presumably bind to this region to more efficiently initiate or enhance the expression of neighbouring genes. The actual protein binding ability of the 621-bp insertion was evaluated using electrophoretic mobility shift assays (EMSAs). Nuclear protein(s) extracted from Tapidor before vernalization were able to bind to some fragments from the middle of the 621-bp *Monkey King* region that contained TATA box motifs (unpublished data). These results suggest that *Monkey King* can bind to specific transcription factors that may initiate or enhance *BnFLC.A10* expression in winter rapeseed cultivars, giving rise to their stronger vernalization requirement.

Our analysis also indicated that *Monkey King* is involved in gene regulation in many different settings in the genome. For example, we found three copies of the sequence GACTGGTT near the 5’ end of *Monkey King* (Figure 3C); this motif is conserved in the upstream region of *Dsg1* (desmoglein1, which encodes desmosomal cadherin) in mice. The motif in *Dsg1* is recognized by GRHL1 (grainyhead-like 1, a homolog of the *Drosophila* gene grainyhead) and increases *Dsg1* expression [49]. Part of the *Monkey King* sequence is transcribed in *Brassica* genomes (http://www.ncbi.nlm.nih.gov, Table 3), and has been identified in the 3' untranslated region of the *WRKY21-1* gene (EU912394). Other transcripts that share high similarity with portions of the *Monkey King* sequence have been found in the expressed sequence tag library of rapeseed (http://www.ncbi.nlm.nih.gov). The presence of these transcripts suggests the existence of a novel gene regulatory mechanism that is similar to the method by which exon shuffling generates new genes [50,51] or overlapping transcripts generate siRNAs to regulate gene expression [52,53]. It is possible that transcripts derived from *Monkey King* might regulate native gene expression through siRNA-induced DNA methylation. MITE activities within *BnFLC.Al0* may have shaped phenotypic diversity and influenced mechanisms of adaptation to diverse climates during the evolutionary process. 

**Table 3:** **20 sequences that show high similarity with *Monkey King *in the *B. napus *EST library**

**Accession**	**Aligned position in Monkey King (bp)**	**Query coverage**	**E value**	**Max identity**	**Description**
CD826040.1	29-586	90%	0	89%	BN25.062J15F011130 BN25 Brassica napus cDNA clone BN25062J15, mRNA sequence
EV022063.1	1-416	67%	3.00E-164	91%	BNSCS2CT_UP_086_C03_19APR2007_027 Brassica napus BNSCS2CT Brassica napus cDNA 5', mRNA sequence
EE567409.1	29-437	66%	5.00E-157	91%	BNZB_UP_149_C07_29SEP2005_059 Brassica napus BNZB Brassica napus cDNA 5', mRNA sequence
ES968675.1	5-416	66%	2.00E-151	90%	BNZB_UP_208_D10_15MAR2006_074 Brassica napus BNZB Brassica napus cDNA 5', mRNA sequence
EE558281.1	1-400	64%	2.00E-151	90%	BNZB_RP_027_G10_28APR2004_068 Brassica napus BNZB Brassica napus cDNA 5', mRNA sequence
EE564397.1	30-613	94%	2.00E-151	84%	BNZB_UP_107_G11_23AUG2004_083 Brassica napus BNZB Brassica napus cDNA 5', mRNA sequence
GT085003.1	140-613	76%	1.00E-143	86%	c08_20na_1j.s 20na Brassica napus cDNA clone c08_20na_1j 5, mRNA sequence
ES956896.1	87-416	53%	8.00E-125	91%	9RDBNGA_UP_157_G11_10MAR2006_083 Brassica napus 9RDBNGA Brassica napus cDNA 5', mRNA sequence
EE567134.1	379-618	38%	4.00E-108	96%	BNZB_UP_144_H11_27SEP2005_081 Brassica napus BNZB Brassica napus cDNA 5', mRNA sequence
EE567253.1	373-618	39%	1.00E-107	95%	BNZB_UP_147_A07_27SEP2005_063 Brassica napus BNZB Brassica napus cDNA 5', mRNA sequence
EE559708.1	379-618	38%	1.00E-107	96%	BNZB_UP_048_D04_11MAY2004_026 Brassica napus BNZB Brassica napus cDNA 5', mRNA sequence
EE566332.1	315-552	38%	2.00E-091	92%	BNZB_UP_133_A11_27SEP2005_095 Brassica napus BNZB Brassica napus cDNA 5', mRNA sequence
ES903789.1	5-203	32%	3.00E-079	93%	BNARO4GH_T3_002_A02_24NOV2006_016 Brassica napus BNARO4GH Brassica napus cDNA 5', mRNA sequence
EE567417.1	390-613	36%	9.00E-055	84%	BNZB_UP_149_D08_29SEP2005_058 Brassica napus BNZB Brassica napus cDNA 5', mRNA sequence
FG554276.1	441-613	27%	7.00E-051	88%	BN18DYSC_UP_016_A09_18FEB2008_079 BN18DYSC Brassica napus cDNA 5', mRNA sequence
EE568964.1	438-613	28%	1.00E-048	86%	BNZB_UP_170_G02_30SEP2005_004 Brassica napus BNZB Brassica napus cDNA 5', mRNA sequence
EV193796.1	438-613	28%	1.00E-043	85%	0091281 Brassica napus Cold acclimation - dark Brassica napus cDNA, mRNA sequence
FG577502.1	539-613	12%	2.00E-021	92%	BN24DYSC_UP_080_D10_8FEB2008_074 BN24DYSC Brassica napus cDNA 5', mRNA sequence

## Conclusions

This study demonstrated that *BnFLC.A10* is the highly likely causative gene underlying *qFT10-4*, which accounted for most flowering time variation in the TN DH population under spring environmental conditions. Comparision of allelic sequences from Tapidor and Ningyou7 revealed the presence of a *Tourist*-like MITE insertion in winter-type cultivar Tapidor. Association analysis among winter- and spring-type rapeseeds revealed that the presence of the *Tourist*-like MITE insertion is very strongly associated with vernalization requirement, and suggested that it appeared after *B. napus* was generated as a product of natural hybridization between *B. rapa* and *B. oleracea*. MITE activity led to genetic and phenotypic diversities among varieties and provided the fuel for evolutionary selection. As a result, winter genotypes may have evolved from spring genotypes; this useful variation has subsequently been used as a genetic resource for the development of winter cultivars enabling worldwide production of rapeseed.

## Methods

### Plant materials

For fine mapping of the *BnFLC.A10* locus, we used 9,000 plants derived from four BC_5_F_1_ individuals: 8y085-1, 8y086-1, 8y086-2 and 8y086-4. TN DH043 (the 43rd line of the TN DH population) was crossed wiht Ningyou7 (semi-winter recurrent parent) and seeds were collected from the F_1_ generation. Plants were then backcrossed with Ningyou7 over five successive generations (BC_1_ to BC_5_). Molecular markers were used to track the Tapidor allele at the *BnFLC.A10* locus in the F1 backcross. The BC_5_F_2_ near-isogenic lines were planted in the spring of 2009 for phenotyping with respect to flowering time. A panel of 79 diverse rapeseed cultivars (Table 1) was used for the association analysis. These cultivars were planted in spring during three successive years (2007–2009) for phenotyping. Climatic conditions during the planting season and geographic features of the planting site were as described previously [36]. The spring rapeseed and *B. rapa* accessions representing nine subspecies that were used to detect the presence of *Monkey King* upstream of *BnFLC.A10* and *BrFLC.A10* are listed in Additional file 2. These accessions were obtained from the National *Brassica* Germplasm Improvement Program (Wagga Wagga, Australia), the Australian Temperate Field Crops Collection (Horsham, Australia), and from the Institute of Vegetables and Flowers, Chinese Academy of Agricultural Sciences (Beijing, China).

### Phenotypic evaluation

Flowering times of the different cultivars used for the association analysis were recorded as the number of days from the day of sowing to the day when 50% of plants in the plot flowered. In the BC_5_F_2_ populations, days to flowering (DTF) were recorded as the number of days from the day of sowing to the day when the first flower opened. The phenotype ‘non-flowering’ was assigned when plants showed no visible buds at autumn harvest in middle-October. Phenotypes of the 79 cultivars used for the association analysis are listed in Table 1.

### Sequencing of *BnFLC.A10* alleles from Tapidor and Ningyou7

A fragment amplified from the Tapidor genome with primer pair “Exon 4-7” (Table 4) was used as a probe to screen the Tapidor BAC library [54]. From 12 BAC clones that contained *BnFLC.A10*, one clone with the code JBnB75D10 was selected and sequenced to obtain the *BnFLC.A10-T* allele. Primers (P4, P5, exon1-2,exon2-4, exon4-7, Table 4)were designed based on the basis of the *BnFLC.A10-T* sequence and used to obtain the sequence of *BnFLC.A10-N*. The amplicons were cloned into a pGEM-T Easy vector (Promega, Madison, WI, USA) and sequenced to determine the *BnFLC.A10-N* sequence. Information on primers and amplified gene regions is provided in Table 4. *BnFLC.A10-N* and *BnFLC.A10-T* sequences were obtained, accession numbers [GenBank: JX901141 and JX901142]. 

**Table 4:** **Sequence information for primers used for polymorphism and *BnFLC.A10 *gene expression analysis**

**Primer name**	**Sequence(5'-3')**
BnFLC.A10 specific primers	
Exon1-2 f	CATCCGTCGCTCTTCTTGTC
Exon1-2 r	GTTGCTTTCCATATCGATCAAG
Exon2-4 f	AACATGATGATGATCTTAAAGCCT
Exon2-4 r	CTCCAGCTGAACCAGGGAAC
Exon4-7 f	CTTGAGGAATCAAATGTCGATAA
Exon4-7 r	CGGAGATTTGTCCTGGTGAG
InDel1 f (P4 f)	GGTTCCTTTTCTTTTCGTTTGGG
InDel1 r (P4 r)	GAAGTAAAGTCGGACAAGAAGG
InDel2 f (P5 f)	CCTTCTTGTCCGACTTTACTTC
InDel2 r (P5 r)	CGTTGCTCCTACTTTGTCTATC
InDel3 (IP1IP2) f	CGTCGCTCTTCTTGTCGTCTC
InDel3 (IP1IP2) r	TATGCATCACAGCGTGTCAAA
InDel4 f	GTGTTCAGCTGTCGCTTCTAT
InDel4 r	CTAACGCTGGCTTTGATCTT
Itr1f	AATACTTCCTGCGAATCTTGTG
Itr1r	AGTTTGCTTCTAAGTCCCCAAT
SSR primers developed from JBnB75D10	
25GTTA f	ACTTTCATCACCATTGCAGACA
25GTTA r	AAGAGCAGCCATTGTATCAGGT
T11 f	TTCCCAAGCTTGCTGGTACT
T11 r	GAGATTTCCCTCGCTTGATG
NIAB009 f	TACGCTAGTGAGAACACCTCCA
NIAB009 r	GCTTTAGCAAGAAAACTCGGAA
q-RT PCR primers	
Prt f	TCCGTCGCTCTTCTTGTCGT
Prt r	GCTGAACCAGGGAACCCACA
actin2 F	CTGTGCCAATCTACGAGGGTTTC
actin2 R	CTTACAATTTCCCGCTCTGCTGT
18S f	GAGTATGGTCGCAAGGCTGAAA
18S r	CGCTCCACCAACTAAGAACGG

### Gene annotation for the BAC sequence

Gene annotation was carried out using the FGENESH program by selection of the organism category “Dicot plants (*A. thaliana*)” and alignment with *A. thaliana* genes. Simple sequences and transposons were identified using RepeatMasker (http://www.repeatmasker.org/, validated 19th September, 2011) followed by manual inspection. We predicted the function of genes that were not aligned with *A. thaliana* orthologs from their conserved domains.

### RNA extraction and q-RT PCR

Plants were grown under long-day conditions (16 h light/8 h dark) at 23°C until they had developed to the six-leaf stage, at which point they were transferred to 4°C for vernalization. *BnFLC.A10* expression was analyzed in plants that had been subjected to a (control), 1, 4 and 7 weeks of vernalization. Total RNA was extracted from plant leaves using TRIzol® reagent (Invitrogen, Carlsbad, California, USA). Total RNA (2 μg) was reverse-transcribed using M-MLV Reverse Transcriptase (Promega). An iQ5 Real-Time PCR Detection System (Bio-Rad, Hercules, CA, USA) was used for quantitative RT-PCR to detect levels of *BnFLC.A10* expression in the two parents. *BnFLC.A10* primers (Prt f/Prt r, Table 4) amplified a 235-bp fragment of the *BnFLC.A10* CDS. Two genes, *actin*2 and 18S rRNA (Table 4), were used to normalize expression levels. Three biological and technical replicates were analyzed.

### Natural variation in *BnFLC.A10*

Allele-specific primers “Itr1f/Itr1r” (Table 4) were used to distinguish the *BnFLC.A10* Tapidor allele from the Ningyou7 *BnFLC.A10* variant in 24 rapeseed cultivars. The PCR products were cloned into a pGEM-T Easy vector (Promega) for sequencing. Plasmid prepared from two to four colonies from each PCR product was sequenced separately to minimize the contribution of polymerase errors to sequence variation.

### Screening of homologous sequences of *Monkey King* in the *B. rapa* genome

To identify homologous sequences, the full length MITE sequence was queried against the *B. rapa* genome in the brassicadb database (http://brassicadb.org/) using BLAST. Results were filtered using an *E* value<1e^-10^ as the cutoff.

## Competing interests

The authors declare that they have no competing interests.

## Authors’ contributions

JH, YL and XZ carried out NIL and segregation population development, gene cloning and flowering time investigations; HR and JW conducted phenotypic and association analysis and detected *Monkey King* in spring rapeseed and *B. rapa* varieties; SD, QX and CL conducted flowering time investigations and genotyping of the segregation population; LF performed the MITE structural analysis; BL conducted BAC (JBnB75D10) sequencing; JH and JM designed and supervised the study, analyzed the data and wrote the paper. All the authors discussed the results and contributed to the manuscript. All authors read and approved the final manuscript.

## Supplementary Material

Additional file 1 Motif prediction of 621bp-MITE. Click here for file

Additional file 2 **Spring rapeseed and *B. rapa *accessions were used for detecting *Monkey King *existence upstream of *BnFLC.A10 *and the orthologous region.** Click here for file
