# Supplementary material for: A Tourist-like MITE insertion in the upstream region of the BnFLC.A10 gene is associated with vernalization requirement in rapeseed (Brassica napus L.)
Source: BMC Plant Biol. 2012 Dec 15;12:238. doi: 10.1186/1471-2229-12-238 (PMC3562271; doi:10.1186/1471-2229-12-238)
Supplement: Additional file 1 — Motif prediction of 621bp-MITE. [file 1471-2229-12-238-S1.pdf]

Additional file 1. Motif prediction of 621bp-MITE

Code	Taxon	Start	Strand	Length	Motif	Function
ARE	*Zea mays*	408	+	6	TGGTTT	cis-acting regulatory element essential for the anaerobic induction
ARE	*Zea mays*	602	-	6	TGGTTT	cis-acting regulatory element essential for the anaerobic induction
Box 4	*Petroselinum crispum*	322	+	6	ATTAAT	part of a conserved DNA module involved in light responsiveness
Box I	*Pisum sativum*	257	+	7	TTTCAAA	light responsive element
CAAT-box	*Brassica rapa*	88	-	5	CAAAT	common cis-acting element in promoter and enhancer regions
CAAT-box	*Hordeum vulgare*	538	-	4	CAAT	common cis-acting element in promoter and enhancer regions
CAAT-box	*Hordeum vulgare*	186	+	4	CAAT	common cis-acting element in promoter and enhancer regions
CAAT-box	*Hordeum vulgare*	606	+	4	CAAT	common cis-acting element in promoter and enhancer regions
CAAT-box	*Arabidopsis thaliana*	116	-	5	CCAAT	common cis-acting element in promoter and enhancer regions
CAAT-box	*Hordeum vulgare*	566	+	4	CAAT	common cis-acting element in promoter and enhancer regions
CAAT-box	*Brassica rapa*	519	-	5	CAAAT	common cis-acting element in promoter and enhancer regions
CAAT-box	*Arabidopsis thaliana*	565	+	5	CCAAT	common cis-acting element in promoter and enhancer regions
CAAT-box	*Glycine max*	115	-	5	CAATT	common cis-acting element in promoter and enhancer regions
CAAT-box	*Arabidopsis thaliana*	605	+	5	CCAAT	common cis-acting element in promoter and enhancer regions
CAAT-box	*Brassica rapa*	155	+	5	CAAAT	common cis-acting element in promoter and enhancer regions
E2Fa	*Nicotiana tabacum*	596	-	8	TTTCCCGC	
GT1-motif	*Arabidopsis thaliana*	145	+	6	GGTTAA	light responsive element
GT1-motif	*Arabidopsis thaliana*	168	+	6	GGTTAA	light responsive element
HSE	*Brassica oleracea*	274	-	9	AAAAAATTTC	cis-acting element involved in heat stress responsiveness
Skn-1_motif	*Oryza sativa*	139	-	5	GTCAT	cis-acting regulatory element required for endosperm expression
Sp1	*Zea mays*	57	-	5.5	CC(G/A)CCC	light responsive element
Sp1	*Oryza sativa*	450	-	6	GGGCGG	light responsive element
Sp1	*Zea mays*	440	+	5	CC(G/A)CCC	light responsive element
Sp1	*Zea mays*	588	-	5.5	CC(G/A)CCC	light responsive element

TATA-box	*Lycopersicon esculentum*	79	+	5	TTTTA	core promoter element around -30 of transcription start
TATA-box	*Glycine max*	175	+	5	TAATA	core promoter element around -30 of transcription start
TATA-box	*Pisum sativum*	196	-	8	TATAAAAT	core promoter element around -30 of transcription start
TATA-box	*Arabidopsis thaliana*	197	-	7	TATAAAA	core promoter element around -30 of transcription start
TATA-box	*Arabidopsis thaliana*	198	-	6	TATAAA	core promoter element around -30 of transcription start
TATA-box	*Arabidopsis thaliana*	199	-	7	TATATAA	core promoter element around -30 of transcription start
TATA-box	*Arabidopsis thaliana*	200	+	4	TATA	core promoter element around -30 of transcription start
TATA-box	*Brassica oleracea*	201	+	7	ATATAAT	core promoter element around -30 of transcription start
TATA-box	*Arabidopsis thaliana*	202	+	11	TATAAATATAAA	core promoter element around -30 of transcription start
TATA-box	*Brassica napus*	206	+	6	ATTATA	core promoter element around -30 of transcription start
TATA-box	*Arabidopsis thaliana*	207	-	5	TATAA	core promoter element around -30 of transcription start
TATA-box	*Arabidopsis thaliana*	208	+	6	TATAAA	core promoter element around -30 of transcription start
TATA-box	*Lycopersicon esculentum*	210	-	5	TTTTA	core promoter element around -30 of transcription start
TATA-box	*Glycine max*	228	+	5	TAATA	core promoter element around -30 of transcription start
TATA-box	*Glycine max*	231	-	5	TAATA	core promoter element around -30 of transcription start
TATA-box	*Brassica napus*	232	+	6	ATTATA	core promoter element around -30 of transcription start
TATA-box	*Arabidopsis thaliana*	233	-	5	TATAA	core promoter element around -30 of transcription start
TATA-box	*Arabidopsis thaliana*	234	+	4	TATA	core promoter element around -30 of transcription start
TATA-box	*Glycine max*	236	+	5	TAATA	core promoter element around -30 of transcription start
TATA-box	*Brassica napus*	250	+	6	ATTATA	core promoter element around -30 of transcription start
TATA-box	*Arabidopsis thaliana*	251	-	5	TATAA	core promoter element around -30 of transcription start
TATA-box	*Arabidopsis thaliana*	252	+	4	TATA	core promoter element around -30 of transcription start
TATA-box	*Arabidopsis thaliana*	265	-	5	TATAA	core promoter element around -30 of transcription start
TATA-box	*Arabidopsis thaliana*	266	+	4	TATA	core promoter element around -30 of transcription start
TATA-box	*Lycopersicon esculentum*	271	+	5	TTTTA	core promoter element around -30 of transcription start
TATA-box	*Zea mays*	279	-	8	TTTAAAAA	core promoter element around -30 of transcription start

TATA-box	*Lycopersicon esculentum*	280	+	5	TTTTA	core promoter element around -30 of transcription start
TATA-box	*Brassica napus*	286	+	6	ATTATA	core promoter element around -30 of transcription start
TATA-box	*Arabidopsis thaliana*	287	-	5	TATAA	core promoter element around -30 of transcription start
TATA-box	*Arabidopsis thaliana*	288	+	4	TATA	core promoter element around -30 of transcription start
TATA-box	*Lycopersicon esculentum*	300	+	5	TTTTA	core promoter element around -30 of transcription start
TATA-box	*Lycopersicon esculentum*	305	+	5	TTTTA	core promoter element around -30 of transcription start
TATA-box	*Lycopersicon esculentum*	308	-	5	TTTTA	core promoter element around -30 of transcription start
TATA-box	*Pisum sativum*	312	-	8	TATAAAAT	core promoter element around -30 of transcription start
TATA-box	*Arabidopsis thaliana*	313	-	7	TATAAAA	core promoter element around -30 of transcription start
TATA-box	*Arabidopsis thaliana*	314	-	6	TATAAA	core promoter element around -30 of transcription start
TATA-box	*Arabidopsis thaliana*	315	-	5	TATAA	core promoter element around -30 of transcription start
TATA-box	*Arabidopsis thaliana*	316	+	4	TATA	core promoter element around -30 of transcription start
TATA-box	*Glycine max*	318	+	5	TAATA	core promoter element around -30 of transcription start
TATA-box	*Glycine max*	321	-	5	TAATA	core promoter element around -30 of transcription start
TATA-box	*Lycopersicon esculentum*	328	-	5	TTTTA	core promoter element around -30 of transcription start
TATA-box	*Brassica oleracea*	332	+	7	ATATAAT	core promoter element around -30 of transcription start
TATA-box	*Arabidopsis thaliana*	333	+	4	TATA	core promoter element around -30 of transcription start
TATA-box	*Glycine max*	335	+	5	TAATA	core promoter element around -30 of transcription start
TATA-box	*Brassica napus*	341	+	6	ATATAT	core promoter element around -30 of transcription start
TATA-box	*Arabidopsis thaliana*	342	+	4	TATA	core promoter element around -30 of transcription start
TATA-box	*Lycopersicon esculentum*	346	+	5	TTTTA	core promoter element around -30 of transcription start
TATA-box	*Arabidopsis thaliana*	355	-	7	TATAAAA	core promoter element around -30 of transcription start
TATA-box	*Arabidopsis thaliana*	356	-	6	TATAAA	core promoter element around -30 of transcription start
TATA-box	*Arabidopsis thaliana*	357	-	5	TATAA	core promoter element around -30 of transcription start
TATA-box	*Arabidopsis thaliana*	358	+	4	TATA	core promoter element around -30 of transcription start
TATA-box	*Lycopersicon esculentum*	368	+	5	TTTTA	core promoter element around -30 of transcription start

TATA-box	*Zea mays*	369	+	8	TTTAAAAA	core promoter element around -30 of transcription start
TATA-box	*Lycopersicon esculentum*	371	-	5	TTTTA	core promoter element around -30 of transcription start
TATA-box	*Lycopersicon esculentum*	377	+	5	TTTTA	core promoter element around -30 of transcription start
TATA-box	*Arabidopsis thaliana*	379	-	9	TAAAAATAA	core promoter element around -30 of transcription start
TATA-box	*Lycopersicon esculentum*	383	+	5	TTTTA	core promoter element around -30 of transcription start
TATA-box	*Arabidopsis thaliana*	385	-	9	TAAAAATAA	core promoter element around -30 of transcription start
TATA-box	*Lycopersicon esculentum*	389	+	5	TTTTA	core promoter element around -30 of transcription start
TATA-box	*Oryza sativa*	395	-	7	TACAAAA	core promoter element around -30 of transcription start
TATA-box	*Arabidopsis thaliana*	396	-	11	TATAAATATAAA	core promoter element around -30 of transcription start
TATA-box	*Daucus carota*	400	-	8	TATAAATA	core promoter element around -30 of transcription start
TATA-box	*Ac*	401	-	7	TATAAAT	core promoter element around -30 of transcription start
TATA-box	*Arabidopsis thaliana*	402	-	6	TATAAA	core promoter element around -30 of transcription start
TATA-box	*Arabidopsis thaliana*	403	-	5	TATAA	core promoter element around -30 of transcription start
TATA-box	*Arabidopsis thaliana*	404	+	4	TATA	core promoter element around -30 of transcription start
TATA-box	*Zea mays*	412	-	8	TTTAAAAA	core promoter element around -30 of transcription start
TATA-box	*Lycopersicon esculentum*	413	+	5	TTTTA	core promoter element around -30 of transcription start
TATA-box	*Zea mays*	414	+	8	TTTAAAAA	core promoter element around -30 of transcription start
TATA-box	*Lycopersicon esculentum*	416	-	5	TTTTA	core promoter element around -30 of transcription start
TATA-box	*Lycopersicon esculentum*	490	+	5	TTTTA	core promoter element around -30 of transcription start
Unnamed__4	*Petroselinum hortense*	441	+	4	CTCC	
Unnamed__4	*Petroselinum hortense*	503	-	4	CTCC	
box E	*Petroselinum crispum*	604	+	9	ACCCATCAAG	

PlantCARE: a database of plant cis-acting regulatory elements and a portal to tools for in silico analysis of promoter sequences.

Lescot, M., Déhais, P., Moreau, Y., De Moor, B., Rouzé,P.,and Rombauts, S.Nucleic Acids Res., Database issue(2002), 30(1):325-327.
