# Supplementary material for: A Tourist-like MITE insertion in the upstream region of the BnFLC.A10 gene is associated with vernalization requirement in rapeseed (Brassica napus L.)
Source: BMC Plant Biol. 2012 Dec 15;12:238. doi: 10.1186/1471-2229-12-238 (PMC3562271; doi:10.1186/1471-2229-12-238)
Supplement: Additional file 2 — Spring rapeseed and B. rapa accessions were used for detecting Monkey King existence upstream of BnFLC.A10 and the orthologous region. [file 1471-2229-12-238-S2.pdf]

Additional file 2. Spring rapeseed and *B. rapa* accessions were used for detecting *Monkey King* existance upstream of *BnFLC.A10* and the orthologous region

Name	Species	*Monkey King* existance
VALTTI	*Brassica rapa*	No
CHOKUREI	*Brassica rapa*	No
CHOY SIM	*Brassica rapa*	No
COW HORN LONG WHITE	*Brassica rapa*	No
CPI 57554	*Brassica rapa*	No
SUFOLA	*Brassica rapa*	No
HOSIN	*Brassica rapa*	No
KAGA	*Brassica rapa*	No
MEXICO G	*Brassica rapa*	No
MIE LOCAL	*Brassica rapa*	No
PI 370743	*Brassica rapa*	No
POLISH	*Brassica rapa*	No
SOLLUX	*Brassica rapa*	No
TORPE	*Brassica rapa*	No
TURNIP EXTRA EARLY PURPLE TOP MILAN	*Brassica rapa*	No
YORK GLOBE	*Brassica rapa*	No
PI 179652	*Brassica rapa*	No
JUGOSLAVSKY	*Brassica rapa*	No
GULLE	*Brassica rapa*	No
JALPAIGURI CL-1	*Brassica rapa*	No
TORIA-ITSA	*Brassica rapa*	No
PUSA KALYANI	*Brassica rapa*	No
CANDLE ATR	*Brassica rapa*	No
FATAPUKUR CL-1	*Brassica rapa*	No
CPI91433	*Brassica rapa subsp. chinensis*	No
SALAT YAPONSKII	*Brassica rapa subsp. chinensis*	No
BI-TCE	*Brassica rapa subsp. pekinensis*	No
PIORBAI	*Brassica rapa subsp. chinensis*	No
K-109	*Brassica rapa subsp. pekinensis*	No
MIBUNA	*Brassica rapa subsp. nipposinica*	No
K-123	*Brassica rapa subsp. pekinensis*	No
K-136	*Brassica rapa subsp. pekinensis*	No
DUNGANSKAYA	*Brassica rapa subsp. pekinensis*	No
MIZUNA	*Brassica rapa subsp. nipposinica*	No
Leielander Waasmunster	*Brassica rapa*	No
CGN 6850	*Brassica rapa*	No
CGN 7222	*Brassica rapa*	No
CGN 7226	*Brassica rapa*	No
Balady	*Brassica rapa*	No
Tsja Sin	*Brassica rapa*	No

Tsja Sin	*Brassica rapa*	No
CGN 20198	*Brassica rapa*	No
CGN 20736	*Brassica rapa*	No
CGN 20738	*Brassica rapa*	No
Rova, svedjerova	*Brassica rapa subsp. rapa*	No
K-4170	*Brassica rapa subsp. oleifera*	No
Pusa Chandrina	*Brassica rapa*	No
Somali Sarisa	*Brassica rapa*	No
Candle	*Brassica rapa*	No
Perviridis	*Brassica rapa*	No
CGN07187	*Brassica rapa*	No
CGN07216	*Brassica rapa*	No
CGN07222	*Brassica rapa*	No
Balady	*Brassica rapa*	No
Tientsin	*Brassica rapa*	No
Xiao qing kou	*Brassica rapa*	No
CGN15218	*Brassica rapa*	No
Jinengu-Kabu	*Brassica rapa*	No
NABAL DE GREILOS	*Brassica rapa subsp. rapa*	No
HRI 6175	*Brassica rapa subsp. pekinensis*	No
NOZAKI EARLY	*Brassica rapa subsp. pekinensis*	No
HRI 8170	*Brassica rapa subsp. rapa*	No
BRA 228	*Brassica rapa subsp. chinensis*	No
Matsushima Shin 2	*Brassica rapa subsp. pekinensis*	No
BRA 981 / 01	*Brassica rapa subsp. pekinensis*	No
Horpacsi Lila	*Brassica rapa subsp. rapa*	No
Rapa sponsa	*Brassica rapa subsp. oleifera*	No
Wase Mibuna	*Brassica rapa subsp. nipposinica*	No
BRA 1302 / 01	*Brassica rapa subsp. pekinensis*	No
BRA 1304 / 01	*Brassica rapa subsp. pekinensis*	No
Esti Naeris	*Brassica rapa subsp. rapa*	No
BRA 1005	*Brassica rapa subsp. oleifera*	No
BRA 41	*Brassica rapa subsp. oleifera*	No
B 046	*Brassica rapa subsp. trilocularis*	No
Pusa Kalyani	*Brassica rapa subsp. dichotoma*	No
CR2867 / 03	*Brassica rapa*	No
CR2881 / 79	*Brassica rapa*	No
CR2901 / 82	*Brassica rapa*	No
Baijian 13	*Brassica rapa* subsp. oleifera	No
Chengduaiyoucai	*Brassica rapa* subsp. oleifera	No
Chiifu	*Brassica rapa* subsp. pekinensis	No
Chunqiuwang	*Brassica rapa* subsp. pekinensis	No
Colt	*Brassica rapa*	No
Denglongzhong	*Brassica rapa* subsp. oleifera	No

Dongkoutianyoucai	*Brassica rapa* subsp. oleifera	No
DS17D	*Brassica rapa*	No
Fengkang70	*Brassica rapa* subsp. pekinensis	No
Hongcaitaierzaozi	*Brassica rapa* var. purpuraria	No
Huabai2	*Brassica rapa* subsp. pekinensis	No
Huangxinwubaicai	*Brassica rapa* subsp. pekinensis	No
Maverick	*Brassica rapa* subsp. oleifera	No
Qingchun	*Brassica rapa* subsp. pekinensis	No
Qingqing	*Brassica rapa* subsp. pekinensis	No
Taiyuanerqing	*Brassica rapa* subsp. pekinensis	No
Teji49huangcaixin	*Brassica rapa* var. parachinensis	No
Tianmenyoucaibai	*Brassica rapa* subsp. oleifera	No
Torch	*Brassica rapa*	No
V02D0181	*Brassica rapa* var. parachinensis	No
V02D0209	*Brassica rapa* var. parachinensis	No
Wulitianyoucai	*Brassica rapa* subsp. oleifera	No
Xinghuayoucai	*Brassica rapa* subsp. oleifera	No
Yuqing101B-4	*Brassica rapa* subsp. pekinensis	No
488youqingcaixinF1	*Brassica rapa* var. parachinensis	No
CB Trilogy	*Brassica napus*	No
CB Telfer	*Brassica napus*	No
GSC 5	*Brassica napus*	No
CB Pilbara	*Brassica napus*	No
CB Trigold	*Brassica napus*	No
CB Tanami	*Brassica napus*	No
Tranby	*Brassica napus*	No
Tarcoola	*Brassica napus*	No
CB Boomer	*Brassica napus*	No
Monty	*Brassica napus*	No
Surpass404CL	*Brassica napus*	No
ATR-Cobbler	*Brassica napus*	No
TornadoTT	*Brassica napus*	No
ThunderTT	*Brassica napus*	No
TawrifficTT	*Brassica napus*	No
RottnestTTC	*Brassica napus*	No
Scoop	*Brassica napus*	No
ATR-Banjo	*Brassica napus*	No
Surpass402CL	*Brassica napus*	No
BLN3343*CO0401	*Brassica napus*	No
ATR-Barra	*Brassica napus*	No
Surpass400	*Brassica napus*	No
CB Argyle	*Brassica napus*	No
SARDI607	*Brassica napus*	No
Surpass501TT	*Brassica napus*	No

FlindersTTC	*Brassica napus*	No
Dunkeld	*Brassica napus*	No
Lantern	*Brassica napus*	No
BLN3343*CO0402	*Brassica napus*	No
Surpass603CL	*Brassica napus*	No
Hyola60	*Brassica napus*	No
Hyola61	*Brassica napus*	No
ATR-Hyden	*Brassica napus*	No
Karoo	*Brassica napus*	No
Wesroona	*Brassica napus*	No
TERI (OO)R9903	*Brassica napus*	No
Purler	*Brassica napus*	No
AG-Outback	*Brassica napus*	No
03-p74-6	*Brassica napus*	No
BLN3347	*Brassica napus*	No
Charlton	*Brassica napus*	No
ATR409	*Brassica napus*	No
Georgie	*Brassica napus*	No
03-p74-3	*Brassica napus*	No
ZY009	*Brassica napus*	No
ATR-Beacon	*Brassica napus*	No
Oscar	*Brassica napus*	No
Grouse	*Brassica napus*	No
BravoTT	*Brassica napus*	No
ATR-Marlin	*Brassica napus*	No
WA050088	*Brassica napus*	No
Nindoo	*Brassica napus*	No
Shiralee	*Brassica napus*	No
BLN2852-03W02	*Brassica napus*	No
95*306-310.3.9	*Brassica napus*	No
BLN2737*CO0203-1	*Brassica napus*	No
Ag-Muster	*Brassica napus*	No
RocketCL	*Brassica napus*	No
RSO94-67 (98-18)	*Brassica napus*	No
ATR-Signal	*Brassica napus*	No
WA050097	*Brassica napus*	No
HurricaneTT	*Brassica napus*	No
Av-Opal	*Brassica napus*	No
BLN1990	*Brassica napus*	No
BLN3614	*Brassica napus*	No
Rivette	*Brassica napus*	No
ATR-Summitt	*Brassica napus*	No
Ripper	*Brassica napus*	No
44Y06	*Brassica napus*	No

Range	*Brassica napus*	No
Yickadee	*Brassica napus*	No
ZY008	*Brassica napus*	No
WA050096	*Brassica napus*	No
WarriorCL	*Brassica napus*	No
Av-Jade	*Brassica napus*	No
Skipton	*Brassica napus*	No
45C75	*Brassica napus*	No
Rainbow	*Brassica napus*	No
Mystic	*Brassica napus*	No
Ding110	*Brassica napus*	No
Qu1104	*Brassica napus*	No
StormTT	*Brassica napus*	No
Barossa	*Brassica napus*	No
Av-Sapphire	*Brassica napus*	No
Wesbarker	*Brassica napus*	No
BLN2762	*Brassica napus*	No
45Y77	*Brassica napus*	No
Zhongshu-ang N0.4 (30920)	*Brassica napus*	No
BLN3303*CR0302	*Brassica napus*	No
46C04	*Brassica napus*	No
Hyola76	*Brassica napus*	No
03-p74-11	*Brassica napus*	No
45C05	*Brassica napus*	No
Zhongyou-za No.8	*Brassica napus*	No
Yu 178	*Brassica napus*	No
46C76	*Brassica napus*	No
05-P36 R	*Brassica napus*	No
46Y78	*Brassica napus*	No
Tatyoon	*Brassica napus*	No
ATR-EYRE	*Brassica napus*	No
Fan189	*Brassica napus*	No
671109	*Brassica napus*	No
GASGOTT	*Brassica napus*	No
Zhongshu-ang N0.4 (30872)	*Brassica napus*	No
P3083	*Brassica napus*	No
Maluka	*Brassica napus*	No
AG-Emblem	*Brassica napus*	No
Ag-Comet	*Brassica napus*	No
Drakkar	*Brassica napus*	No
Fan028	*Brassica napus*	No
Zhongyou 821	*Brassica napus*	No
44C73	*Brassica napus*	No
Av-Ruby	*Brassica napus*	No

Fan023	*Brassica napus*	No
ZY013	*Brassica napus*	No
ZY002	*Brassica napus*	No
Hyola50	*Brassica napus*	No
ZY007	*Brassica napus*	No
Ag-Spectrum	*Brassica napus*	No
P624	*Brassica napus*	No
ZY003	*Brassica napus*	No
Wesreo	*Brassica napus*	No
N001-28-246-5-4	*Brassica napus*	No
06-P71-2	*Brassica napus*	No
Cescaljarni repka	*Brassica napus*	No
ZY012	*Brassica napus*	No
Ding474	*Brassica napus*	No
Lisora	*Brassica napus*	No
P617	*Brassica napus*	No
ZY001	*Brassica napus*	No
Fan168	*Brassica napus*	No
Wesway	*Brassica napus*	No
ZY015	*Brassica napus*	No
ZY005	*Brassica napus*	No
691198	*Brassica napus*	No
06-P71-1	*Brassica napus*	No
03-p74-4	*Brassica napus*	No
Wesbell	*Brassica napus*	No
04-p34	*Brassica napus*	No
Marnoo	*Brassica napus*	No
ZY010	*Brassica napus*	No
Mutu-98-1	*Brassica napus*	No
Tower-98-21	*Brassica napus*	No
ZY004	*Brassica napus*	No
ZY014	*Brassica napus*	No
Major	*Brassica napus*	No
ZY016	*Brassica napus*	No
Chon nam	*Brassica napus*	No
05-P71-11	*Brassica napus*	No
BLN3614	*Brassica napus*	No
BLN2762	*Brassica napus*	No
Range	*Brassica napus*	No
Surpass400	*Brassica napus*	No
Ag-Outback	*Brassica napus*	No
